# Sodium Bicarbonate and Non-HLA Autoantibody or HLA Antibody Trajectories in Long-term Kidney Transplant Recipients With Metabolic Acidosis: A Post Hoc Analysis of the Preserve-Transplant Randomized Controlled Trial

**DOI:** 10.1097/TXD.0000000000002022

**Published:** 2026-11-06

**Authors:** Alexander Ritter, Jakob Nilsson, Erin West, Christian Kuhn, Carsten A. Wagner, Thomas Schachtner, Nilufar Mohebbi

**Affiliations:** 1 Division of Nephrology, University Hospital Zurich, Zurich, Switzerland.; 2 Division of Nephrology and Transplantation Medicine, HOCH Cantonal Hospital St.Gallen, St. Gallen, Switzerland.; 3 Department of Immunology, University Hospital Zurich, Zurich, Switzerland.; 4 Biostatistics at Clinical Trials Unit, HOCH Cantonal Hospital St.Gallen, St. Gallen, Switzerland.; 5 Institute of Physiology and Zurich Kidney Center, University of Zurich, Zurich, Switzerland.; 6 Praxis und Dialysezentrum Zürich-City AG, Zurich, Switzerland.

## Abstract

**Background.:**

Long-term kidney allograft loss remains a major challenge, driven in part by chronic allograft injury, microvascular inflammation, and antibody-mediated rejection. Beyond HLA donor-specific antibodies, non-HLA autoantibodies such as angiotensin II type 1 receptor (AT1R) and endothelin receptor type A (ETAR) antibodies may contribute. Metabolic acidosis, common after transplantation, has been linked to inflammatory activation, endothelial dysfunction, and upregulation of angiotensin II, aldosterone, and endothelin-1 pathways. Together, these findings raise the hypothesis that metabolic acidosis may contribute to chronic alloimmune and profibrotic injury by amplifying vasculopathy, antibody-mediated microvascular inflammation, and consequently long-term graft dysfunction.

**Methods.:**

This post hoc analysis used prospectively collected data and biobank samples from a randomized, placebo-controlled trial of oral sodium bicarbonate in stable long-term kidney transplant recipients with metabolic acidosis (Preserve-Transplant Study). Non-HLA autoantibodies (AT1R-, ETAR-Abs) and HLA antibodies were measured at baseline and 24 mo, alongside allograft function, acid–base parameters, and immunosuppression.

**Results.:**

AT1R and ETAR antibodies were highly prevalent in the study population. We found no evidence of differences in autoantibody levels between the sodium bicarbonate and control groups over 24 mo. Similarly, donor-specific antibodies and cumulative mean fluorescence intensity (MFI) of overall HLA antibodies did not differ between treatment groups. Furthermore, baseline non-HLA autoantibody levels or positivity for both AT1R- and ETAR-Abs were not associated with estimated glomerular filtration rate (eGFR) slope, whereas cumulative MFI of overall HLA antibodies and donor-specific antibodies were associated with eGFR slope.

**Conclusions.:**

In this post hoc analysis of a randomized controlled trial with stable long-term kidney transplant recipients, we found no evidence to suggest differences in antibody levels of non-HLA autoantibodies (AT1R- and ETAR-Abs) or cumulative MFI of HLA antibodies, including formation of donor-specific antibodies, between the sodium bicarbonate and control groups over 24 mo. Baseline non-HLA autoantibodies were not associated with eGFR slope. These exploratory findings require confirmation in larger prospective studies.

## INTRODUCTION

Despite substantial advances in the management of kidney transplant recipients (KTRs) and improved allograft survival, long-term graft attrition remains a major challenge. Histologic evidence of both immunologic and nonimmunologic damage is detectable in a large proportion of grafts after 10 y.^1,2^ Acute and chronic-active antibody-mediated rejection (AMR) constitute central drivers of microvascular inflammation (MVI) and progressive allograft failure.^3^ AMR is typically mediated by donor-specific antibodies (DSA) targeting endothelial donor HLA.

However, DSA are not consistently detectable in all patients with histologic features of AMR. Reflecting this, the current Banff 2022 classification recognizes “DSA- and C4d-negative microvascular inflammation” as a descriptive phenotype that remains insufficiently understood.^4^ One possible explanation involves antibodies directed against non-HLA auto- and alloantigens. In particular, autoantibodies targeting the angiotensin II type 1 receptor (AT1R) and endothelin receptor type A (ETAR) have been implicated in MVI and graft injury in the absence of HLA-DSA.^5,6^ Supporting this, a recent study demonstrated elevated levels of AT1R- and ETAR-antibodies (Abs) in patients with molecular AMR.^7^ However, whether these non-HLA autoantibodies are causal or arise secondary to preexisting or ongoing endothelial injury remains unresolved.

Metabolic acidosis (MA) is a frequent complication in KTRs.^8-10^ Beyond its association with endothelial dysfunction,^11^ MA has been linked to activation of multiple immune pathways, including proinflammatory cytokines by macrophages and renal tubular epithelial cells, as well as stimulation of the complement system, neutrophils, and dendritic cells.^12-16^ Evidence from other disease contexts further supports the immunomodulatory effects of acidic stress. In solid tumors, acidic stress enhances interferon gamma–induced programmed death-ligand 1 expression on aggressive cancer cells, facilitating immune escape.^17^ Similarly, urine-derived stem cells exposed to acidic conditions secrete increased levels of immunomodulatory cytokines in rheumatoid arthritis.^18^ These observations suggest that chronic MA may promote a proinflammatory microenvironment characterized by enhanced innate immune activation, antigen presentation, and T cell–B cell interactions. Such conditions may lower the threshold for alloimmune activation and thereby facilitate downstream processes, including alloantibody formation and MVI.

Complementing these immune effects, MA also stimulates renal production of key paracrine mediators, including angiotensin II, aldosterone, and endothelin-1 (ET-1).^19-21^ While these responses augment net acid excretion, they also promote adverse hemodynamic and profibrotic effects, particularly through aldosterone. ET-1 may further exacerbate graft injury by driving inflammation, fibrosis, podocyte effacement, and intrarenal angiotensin II amplification.^22-24^ Supporting this, a recent study in KTRs with MA demonstrated a partial reversal of gene expression related to oxidative stress by alkali therapy.^25^ A small biopsy-based analysis indicated a trend toward a higher burden of inflammatory and chronic histological graft lesions with MA.^26^ Together, these findings raise the hypothesis that sustained MA not only reflects impaired tubular acid handling but may actively contribute to chronic alloimmune injury by fostering a proinflammatory and profibrotic milieu.

Collectively, these observations raise the possibility that MA and its downstream hormonal effects on renal tissue interact with both HLA- and non–HLA-mediated pathways to exacerbate MVI and chronic allograft injury, linking nonimmunologic and alloimmune mechanisms in graft dysfunction.

The Preserve-Transplant Study (PTS) is a placebo-controlled, multicenter, randomized clinical trial that evaluated the effect of alkali therapy on estimated glomerular filtration rate (eGFR) in KTRs.^27,28^ While no benefit of alkali therapy on eGFR decline was observed, the impact of MA and its correction on post-transplant humoral immune responses has not been investigated. We therefore aimed to specifically explore whether treatment of MA with sodium bicarbonate (SB) reduces the levels of non-HLA autoantibodies (AT1R-, ETAR-) and cumulative MFI of HLA antibodies, including DSA and their new-onset formation, in stable KTRs over a 2-y treatment period within this randomized controlled trial (RCT) framework.

## PATIENTS AND METHODS

### Hypothesis

This study evaluates the hypothesis that SB therapy, compared with placebo, is associated with fewer non-HLA autoantibodies (AT1R-, ETAR-) and HLA antibodies in KTRs with MA over a 2-y treatment period.

### Study Design and Study Population

This secondary (post hoc) analysis used prospectively collected data and biobank samples from participants enrolled at the University Hospital Zurich, Switzerland, in a randomized, multicenter, single-blind (patients), placebo-controlled trial (PTS). Inclusion criteria were age ≥18 y, ≥12 mo post-transplantation, eGFR 15–89 mL/min/1.73 m^2^, stable allograft function in the preceding 6 mo (<15% change in serum creatinine), and MA defined as serum bicarbonate ≤22 mmol/L. Patients with major electrolyte disturbances or receiving medications with substantial effects on acid–base homeostasis were excluded; full eligibility criteria have been reported previously.^27,28^ The analysis included all participants in the modified intention-to-treat (ITT) population (all randomized Zurich-center patients who completed a baseline visit and received the study intervention). Ethical approval was granted by the Cantonal Ethics Committee (2016–02012), and the study was conducted in accordance with the Declaration of Helsinki. Written informed consent was obtained from all participants.

All patients were followed at a single center with a largely uniform initial maintenance protocol of a calcineurin inhibitor (CNI; tacrolimus or cyclosporine) and mycophenolic acid. Corticosteroids were discontinued at 6 mo post-transplantation in immunologically standard-risk recipients with an uneventful course. They were continued beyond month 6 post-transplantation in patients with preformed DSA against HLA class II (DR/DQ) or other immunological high risk, or where an independent indication existed, and were reinitiated following biopsy-proven T cell–mediated rejection or AMR or the development of de novo DSA. Corticosteroid status was documented at enrollment.

### Study Procedures

Patients were randomized 1:1 to receive oral SB or placebo at an initial daily dose of 1500 mg (<70 kg body weight) or 3000 mg (≥70 kg). At the week-2 titration visit, the daily dose was increased by 1500 mg (to 3000 or 4500 mg) if serum bicarbonate remained ≤22 mEq/L. Serum creatinine, eGFR, trough levels of CNIs and mechanistic (formerly mammalian) target of rapamycin (mTOR) inhibitors, and spot urine albuminuria were assessed every 3 mo. Primary trial data were recorded in a secure, web-based data management system (SecuTrial). Measurements of HLA antibodies and non-HLA autoantibodies (AT1R-, ETAR-Abs) were obtained from biobank samples at baseline and at 24 mo when not otherwise available. The time difference from baseline visit was −32.9 d (SD 78.8, median 0) and at 24 mo −12.5 d (SD 47.1, median 0). HLA antibodies were measured by Luminex (One Lambda) in the Laboratory of Transplantation Immunology at the Department of Immunology of the University Hospital Zurich, Switzerland. Anti-HLA antibodies were reported as mean fluorescence intensity (MFI). The intralaboratory coefficient of variation (CV) was typically <20% for beads with an MFI >1000. Enzyme-linked immunosorbent assay measurements for non-HLA autoantibodies were performed at the CellTrend GmbH laboratory in Luckenwalde, Germany, with manufacturer-reported intra-assay CVs of 3.9% for AT1R-Abs and 6.3% for ETAR-Abs, and the corresponding interassay CVs were 5.1% and 8.3%, respectively.^29,30^

### Outcomes

The primary outcomes were differences between treatment groups (SB versus placebo) over 2 y in: (i) the presence and kinetics of non-HLA autoantibodies (AT1R-, ETAR-Abs) at baseline and 24 mo; (ii) the development of new-onset (de novo) donor-specific HLA antibodies (DSA) during the trial period; (iii) the cumulative MFI of DSA (preformed and/or de novo); and (iv) the cumulative MFI of overall HLA antibodies.

Secondary outcomes included changes in eGFR assessed by the Chronic Kidney Disease Epidemiology Collaboration formula^31^ and albuminuria (spot urine) in relation to levels of non-HLA autoantibodies (AT1R-, ETAR-Abs), cumulative MFI of DSA or overall HLA antibodies. Venous blood pH, serum bicarbonate, and immunosuppressive therapy were compared between groups.

### Statistics

Analyses were performed in a modified ITT population of n = 131 participants from Zurich. Categorical variables are presented as frequencies and percentages; continuous variables are presented as mean (SD). Between-group comparisons were analyzed using Fisher’s exact test for categorical outcomes and the Wilcoxon rank-sum test for continuous outcomes.

For the non-HLA autoantibodies AT1R-Ab and ETAR-Ab, values were capped at 40 U/mL because the CellTrend GmbH ELISA standard curve is validated only up to this concentration. In a sensitivity analysis, samples with optical densities exceeding this range were assigned extrapolated values derived from the standard curve; these measurements were considered semiquantitative and used for this analysis only.

Linear regression models assessed treatment effects on 24-mo antibody levels, adjusting for baseline antibody levels, age, sex, and immunosuppression type. Effect modification by sex and immunosuppression type was evaluated using interaction terms; however, the study was not powered for formal interaction testing, and these analyses were considered exploratory.

New-onset DSA development was defined as the presence of any de novo antibody with a first detection date falling after the patient’s baseline visit. Cumulative MFI was defined as the sum of MFI values across all detected antibodies (DSA, overall HLA antibodies). An MFI threshold of ≥500 was used to define HLA-Ab positivity, including DSA. The eGFR slope for each patient was calculated by least-squares linear regression of all available eGFR measurements, including baseline. Associations between kidney function trajectories and baseline antibody levels were assessed using Spearman correlations.

No imputation of missing data was performed.

Given the exploratory nature of this post hoc analysis, no adjustment for multiple comparisons was applied, and the exploratory results should be interpreted with caution. Analyses were performed in R version 4.5.0.^32^

## RESULTS

### Study Population

The study flowchart of the modified ITT population comprising 131 subjects (Zurich cohort) is illustrated in **Figure S1 (SDC**, https://links.lww.com/TXD/A918). Between June 12, 2017, and July 10, 2019, 66 patients were randomized to SB and 65 to placebo, with 24 mo of follow-up and the last patient visit in July 2021. One hundred five patients finished the trial regularly with available data at both time points, baseline and 24 mo.

Baseline characteristics were similar between groups (Table 1). Mean age was 53.2 (14.4) y; 65.6% of patients were male and 87.8% Caucasian. Repeat transplantation occurred in 9.9%, and more than half received a deceased donor graft. Mean time since transplantation was 9.0 (8.1) y. DSA were reported in 39.0% and prior rejection in 22.9% of patients. CNI-based immunosuppression was used in >90%, with mycophenolate mofetil or mycophenolic acid as the predominant antimetabolites (91.5%). Approximately one-third received corticosteroids. Allograft function and acid–base parameters were comparable between groups.

**TABLE 1.** Baseline characteristics of the intention-to-treat population

		Treatment group	
Characteristic	Overall N = 131^*a*^	Sodium bicarbonate N = 66^*a*^	Placebo N = 65^*a*^
Age, y	53.2 (14.4)	53.5 (13.5)	53.0 (15.3)
Sex			
Female	45 (34.4%)	23 (34.8%)	22 (33.8%)
Male	86 (65.6%)	43 (65.2%)	43 (66.2%)
Race or ethnicity			
Caucasian	115 (87.8%)	56 (84.8%)	59 (90.8%)
Asian	5 (3.8%)	3 (4.5%)	2 (3.1%)
African	2 (1.5%)	1 (1.5%)	1 (1.5%)
Hispanic	3 (2.3%)	2 (3.0%)	1 (1.5%)
Other	6 (4.6%)	4 (6.1%)	2 (3.1%)
Cause of end-stage renal disease			
Hereditary	26 (19.8%)	11 (16.7%)	15 (23.1%)
Glomerulopathy	50 (38.2%)	27 (40.9%)	23 (35.4%)
Tubulointerstitial kidney disease	5 (3.8%)	3 (4.5%)	2 (3.1%)
Diabetic nephropathy	3 (2.3%)	2 (3.0%)	1 (1.5%)
Hypertensive nephropathy	5 (3.8%)	3 (4.5%)	2 (3.1%)
Others	42 (32.1%)	20 (30.3%)	22 (33.8%)
Time since transplantation, y	9.1 (8.1)	9.5 (9.0)	8.7 (7.2)
Number of transplantations			
1	118 (90.1%)	61 (92.4%)	57 (87.7%)
2	12 (9.2%)	4 (6.1%)	8 (12.3%)
3	1 (0.8%)	1 (1.5%)	0 (0.0%)
Repeat transplantation	13 (9.9%)	5 (7.6%)	8 (12.3%)
Transplant from living donor	54 (41.2%)	29 (43.9%)	25 (38.5%)
ABOi	3 (2.3%)	2 (3.0%)	1 (1.5%)
Transplant from deceased donor	77 (58.8%)	37 (56.1%)	40 (61.5%)
History of transplant rejection	30 (22.9%)	13 (19.7%)	17 (26.2%)
Donor-specific HLA antibodies (DSA)	46 (39.0%)	26 (42.6%)	20 (35.1%)
Donor-specific HLA antibodies (DSA) >1	25 (19.2%)	12 (18.2%)	13 (20.3%)
Cumulative MFI of DSA	1911.7 (5035.9)	1989.2 (5015.0)	1828.7 (5101.5)
Pretransplant DSA	22 (16.8%)	15 (15.2%)	12 (18.5%)
Pretransplant peak MFI of DSA	2432 (2225)	2705 (2088)	2205 (2400)
HLA antibodies	118 (100.0%)	61 (100.0%)	57 (100.0%)
Cumulative MFI of HLA antibodies	26 445.7 (50 779.5)	21 721.8 (28 554.1)	31 501.2 (66 809.1)
Primary immunosuppressant			
Tacrolimus	89 (67.9%)	43 (65.2%)	46 (70.8%)
Cyclosporin	32 (24.4%)	18 (27.3%)	14 (21.5%)
mTOR inhibitor	7 (5.3%)	5 (7.6%)	2 (3.1%)
Belatacept	3 (2.3%)	0 (0.0%)	3 (4.6%)
Tacrolimus trough level, µg/L	5.7 (1.9)	5.7 (1.9)	5.7 (1.9)
Ciclosporin trough level, µg/L	67.6 (47.3)	74.2 (60.6)	59.1 (19.5)
mTOR inhibitor trough level, µg/L	5.4 (1.9)	5.3 (2.1)	5.6 (1.8)
Mycophenolate mofetil or mycophenolic acid	119 (91.5%)	61 (93.8%)	58 (89.2%)
Azathioprine	9 (6.9%)	4 (6.2%)	5 (7.7%)
Corticosteroids	43 (33.1%)	20 (30.8%)	23 (35.4%)
Estimated GFR^*b*^, mL/min/1.73 m^2^	50.7 (15.7)	51.2 (15.6)	50.2 (15.9)
Serum bicarbonate, mmol/L	21.6 (2.5)	21.3 (2.7)	21.8 (2.4)
Venous blood pH	7.38 (0.05)	7.38 (0.05)	7.38 (0.05)
Urine protein-to-creatinine ratio, mg/g	271.7 (368.0)	241.1 (251.2)	301.9 (454.8)
Urine albumin-to-creatinine ratio, mg/g	161.2 (304.9)	132.4 (214.0)	189.5 (373.1)

^*a*^Data are presented as mean (SD) or n (%).

^*b*^Estimated with the Chronic Kidney Disease Epidemiology Collaboration equation.

DSA, donor-specific antibodies; GFR, glomerular filtration rate; MFI, mean fluorescence intensity; mTOR, mechanistic (formerly mammalian) target of rapamycin.

### Primary Outcomes

#### Non-HLA Autoantibodies (AT1R-, ETAR-Abs)

Non-HLA autoantibodies could be determined in all study participants at baseline except 1, due to insufficient sample material (**Figure S1, SDC,** https://links.lww.com/TXD/A918). The prevalence of positive non-HLA autoantibody status (≥17 U/mL) at baseline was high (**Table S1, SDC,** https://links.lww.com/TXD/A918), with 58% being positive for AT1R- and 59% for ETAR-Abs. A Fisher’s exact test showed no relevant differences between groups for categories (normal <10 U/mL; risk 10–<17 U/mL; positive ≥17 U/mL) in both AT1R- and ETAR-Ab status.

The kinetics of absolute AT1R- and ETAR-Ab levels over the study duration are illustrated in Figure 1A and B and Table 2. While baseline levels were higher for both AT1R- and ETAR-Abs in the SB group, the change over the study duration did not show differences between groups.

**TABLE 2.** Kinetics: absolute titers of AT1R and ETAR antibodies (patients with both timepoints)

Characteristic	Overall^*a*^ N = 105	Sodium bicarbonate^*a*^ N = 54	Placebo^*a*^ N = 51	*P* ^*b*^
Time between measurements, mo	25.4 (3.0)	25.7 (3.0)	25.1 (3.0)	0.13
AT1R Ab at baseline, U/mL	21 (10)	23 (10)	19 (9)	0.015
AT1R Ab at 24 mo, U/mL	22 (10)	23 (10)	20 (9)	0.064
AT1R Ab change, U/mL	0 (7)	0 (8)	1 (5)	0.10
ETAR Ab at baseline, U/mL	21 (9)	23 (10)	19 (9)	0.014
ETAR Ab at 24 mo, U/mL	22 (9)	24 (10)	20 (8)	0.086
ETAR Ab change, U/mL	1 (7)	1 (8)	2 (6)	0.051

^*a*^Mean (SD).

^*b*^Wilcoxon rank-sum test.

Ab, antibody; AT1R, angiotensin II type 1 receptor; ETAR, endothelin type A receptor.

**FIGURE 1.**
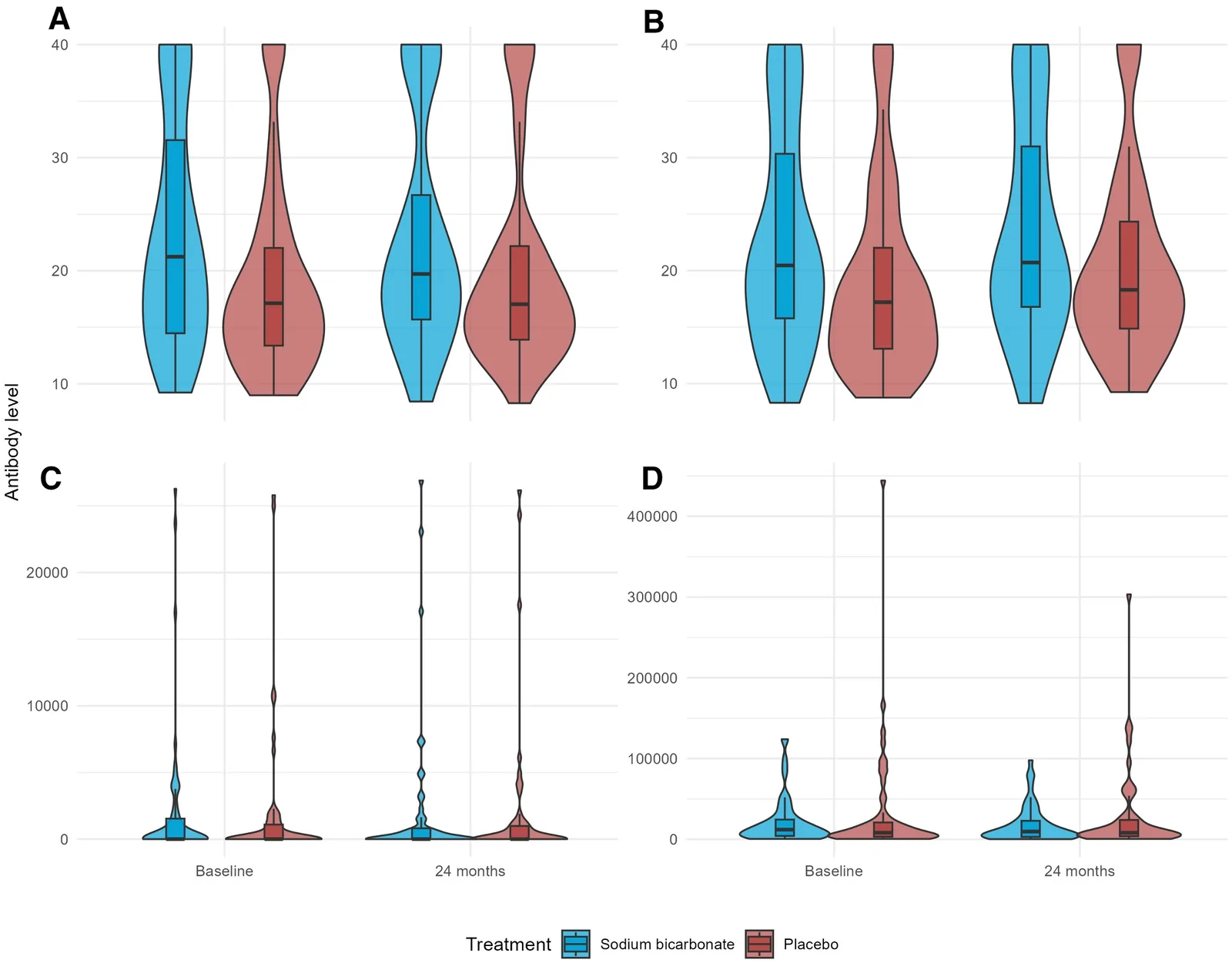
Levels of non-HLA autoantibodies directed against angiotensin II type 1 receptor (AT1R) or endothelin type A receptor (ETAR) (U/mL) and mean fluorescence intensity (MFI) of donor-specific antibodies (DSA) and overall HLA antibodies over the study duration (baseline, 24 mo): AT1R-antibodies (Abs) (A), ETAR-Abs (B), DSA (C), HLA antibodies (D).

SB treatment was not associated with 24-mo AT1R- or ETAR-Ab levels in either baseline-adjusted or fully adjusted models (Table 3). After adjustment for baseline antibody level, age, sex, and immunosuppression type, the estimated treatment effects were small and not statistically significant (AT1R-Abs: *β* = −0.14, 95% confidence interval [CI], −3.25 to 2.98; *P* = 0.93; ETAR-Abs: *β* = 0.39, 95% CI, −2.73 to 3.51; *P* = 0.81).

**TABLE 3.** Linear regression models for 24-mo AT1R and ETAR antibody levels

	AT1R-Ab at 24 mo			ETAR-Ab at 24 mo		
Predictor	*β*	95% CI	*P*	*β*	95% CI	*P*
Baseline model						
Sodium bicarbonate	−0.37	−3.56 to 2.83	0.82	0.14	−3.07 to 3.35	0.93
Adjusted model						
Sodium bicarbonate	−0.14	−3.25 to 2.98	0.93	0.39	−2.73 to 3.51	0.81
Age, y	−0.04	−0.15 to 0.07	0.48	−0.02	−0.13 to 0.08	0.68
Sex (male)	3.78	0.59-6.97	0.02	4.6	1.4-7.81	0.01
Non–CNI-based immunosuppression^*a*^	−5.66	−11.39 to 0.07	0.05	−3.6	−9.34 to 2.13	0.22

^*a*^Mechanistic (formerly mammalian) target of rapamycin inhibitors/belatacept.

Ab, antibody; AT1R, angiotensin II type 1 receptor; CI, confidence interval; CNI, calcineurin inhibitor; ETAR, endothelin type A receptor.

Sensitivity analyses also applying extrapolated non-HLA autoantibody levels (>40 U/mL) without cap at 40 U/mL confirmed the reported results (**Tables S2 and S3, SDC,** https://links.lww.com/TXD/A918).

Furthermore, as shown in **Table S4 (SDC**, https://links.lww.com/TXD/A918), there was no evidence of interaction between treatment and sex or immunosuppression.

Regarding the change of category, more patients improved in the SB group for AT1R-Ab, while slightly more patients worsened for ETAR-Ab in the placebo group; however, no statistical significance was identified (**Table S5 and Figures S2 and S3, SDC,** https://links.lww.com/TXD/A918). Neither the Bland–Altman analysis (**Figure S4, SDC,** https://links.lww.com/TXD/A918) nor the comparison of baseline characteristics (**Tables S6 and S7, SDC,** https://links.lww.com/TXD/A918) identified a distinct group of patients or a consistent pattern of systematic change in antibody levels.

Collectively, these post hoc analyses provided no evidence to suggest an effect of SB treatment on non-HLA autoantibody levels over 24 mo.

#### HLA Antibodies

##### Presence of DSA

As shown in Table 4, the development of new-onset DSA—a rare event—did not differ between groups from baseline to 24-mo follow-up. A Fisher’s exact test was performed to compare the proportion developing new-onset DSA over the 24-mo study period between treatment groups. A sensitivity analysis, including only patients without reported DSA at baseline and with 24-mo follow-up data (n = 63), did not alter this finding.

**TABLE 4.** Development of new-onset (de novo) donor-specific HLA antibodies (DSA) during the trial period (0, 24 mo) and cumulative MFI of preformed/de novo DSA and HLA antibodies (0, 24 mo)

Development of DSA	Overall N = 105^*a*^	Sodium bicarbonate N = 54^*a*^	Placebo N = 51^*a*^	*P* ^*b*^
New-onset DSA (0–24 mo)	4 (3.8%)	3 (5.6%)	1 (2.0%)	0.6
Patients without DSA at baseline	1 (1.6%)	1 (3.1%)	0 (0%)	>0.9
Patients with DSA at baseline	3 (7.1%)	2 (9.1%)	1 (5.0%)	>0.9
Cumulative MFI	Overall N = 105^*c*^	Sodium bicarbonate N = 54^*c*^	Placebo N = 51^*c*^	*P* ^*d*^
DSA				
Cumulative MFI at baseline	2055 (5295)	2066 (5286)	2044 (5357)	0.8
Cumulative MFI at 24 mo	2034 (5381)	2046 (5342)	2023 (5474)	>0.9
Change in cumulative MFI	−21 (2971)	−20 (3159)	−21 (2790)	0.4
DSA: Symmetrical % change in cumulative MFI	−1 (63)	0 (73)	−2 (52)	0.6
HLA antibodies				
Cumulative MFI at baseline	27 189 (52 574)	21 146 (26 463)	33 587 (70 183)	0.7
Cumulative MFI at 24 mo	23 290 (39 967)	18 096 (22 573)	28 790 (52 177)	0.6
Change in cumulative MFI	−3898 (18 724)	−3050 (11 585)	−4797 (24 195)	0.6
HLA Abs: Symmetrical % change in cumulative MFI	−10 (70)	−21 (70)	1 (69)	0.10

^*a*^n (%).

^*b*^Fisher’s exact test.

^*c*^Mean (SD).

^*d*^Wilcoxon rank-sum test.

DSA, donor-specific antibodies; MFI, mean fluorescence intensity.

##### Cumulative MFI of DSA and Overall HLA Antibodies (DSA + Non-DSA)

Cumulative MFI of DSA and overall HLA antibodies (DSA + non-DSA) are illustrated in Figure 1C and D. Both were compared between treatment groups at baseline, at 24 mo, and as change from baseline (Table 4) using the Wilcoxon rank-sum test. No statistically significant differences between groups were observed for cumulative MFI at any time point or for changes from baseline. Likewise, the symmetrical percentage change in cumulative MFI from baseline to 24 mo did not differ significantly between groups for either DSA or overall HLA antibodies.

Furthermore, 24-mo MFI of either DSA or overall HLA antibodies did not differ by SB treatment in baseline-adjusted or fully adjusted models (Table 5).

**TABLE 5.** Linear regression models for 24-mo DSA and HLA antibody MFI

	DSA MFI at 24 mo			HLA MFI at 24 mo		
Predictor	*β*	95% CI	*P*	*β*	95% CI	*P*
Baseline model						
Sodium bicarbonate	3.97	−1120.67 to 1128.62	0.99	−1691.42	−6379.61 to 2996.78	0.48
Adjusted model						
Sodium bicarbonate	−54.31	−1187.93 to 1079.32	0.92	−2052.47	−6747.67 to 2642.72	0.39
Age, y	−20.49	−60.37 to 19.39	0.31	−96.89	−261.93 to 68.15	0.25
Sex (male)	−530.96	−1720.31 to 658.39	0.38	−3735.83	−8623.51 to 1151.84	0.13
Non–CNI-based immunosuppression^*a*^	747.7	−1385.93 to 2881.32	0.49	50.6	−8761.18 to 8862.39	0.99

^*a*^Mechanistic (formerly mammalian) target of rapamycin inhibitors/belatacept.

CI, confidence interval; CNI, calcineurin inhibitor; DSA, donor-specific antibodies; MFI, mean fluorescence intensity.

Taken together, these post hoc analyses provide no evidence to suggest differences in DSA or overall HLA antibody levels according to SB treatment over 24 mo in KTRs with MA.

### Secondary Outcomes

#### Kidney Function and Antibody Status

Moreover, we studied the eGFR slope and baseline albumin-to-creatinine ratio (urine albumin-to-creatinine ratio) in spot urine depending on the cumulative MFI of DSA (preformed/de novo), cumulative MFI of HLA antibodies (including DSA), and AT1R-/ETAR-Ab levels at baseline. A decline in renal function (eGFR slope) was weakly but significantly associated with the cumulative MFI of DSA and more strongly with the cumulative MFI of HLA antibodies. In contrast, no association was observed between the eGFR slope and non-HLA autoantibodies (AT1R-Ab, ETAR-Ab) in our study population (Table 6; Figure 2). AT1R- and ETAR-Ab levels were highly correlated, indicating substantial collinearity (Table 6). Patients with both AT1R and ETAR ≥17 U/mL at baseline (n = 62) did not show a significantly different eGFR slope compared with those without both elevated (−1.7 [5.8] versus −1.7 [5.2]; *P* = 0.5). Using a ≥10 U/mL threshold yielded similar results (*P* = 0.8). No association of any antibody was identified with urine albumin-to-creatinine ratio.

**TABLE 6.** Spearman correlations between eGFR slope/uACR and baseline antibodies

Comparison	n	Spearman*ρ*	*P*
eGFR slope vs cumulative MFI DSA	116	−0.183	0.049
eGFR slope vs cumulative MFI HLA	116	−0.263	0.004
eGFR slope vs AT1R-Ab level	119	0.045	0.628
eGFR slope vs ETAR-Ab level	119	0.055	0.551
Baseline uACR vs cumulative MFI DSA	116	0.216	0.020
Baseline uACR vs cumulative MFI HLA	116	0.112	0.231
Baseline uACR vs AT1R-Ab level	128	−0.062	0.489
Baseline uACR vs ETAR-Ab level	128	−0.025	0.780
AT1R- vs ETAR-Ab level	119	0.929	<0.001

AT1R-Ab, angiotensin II type 1 receptor antibody; DSA, donor-specific antibody; eGFR, estimated glomerular filtration rate; ETAR-Ab, endothelin type A receptor antibody; MFI, mean fluorescence intensity; uACR, urine albumin-to-creatinine ratio.

**FIGURE 2.**
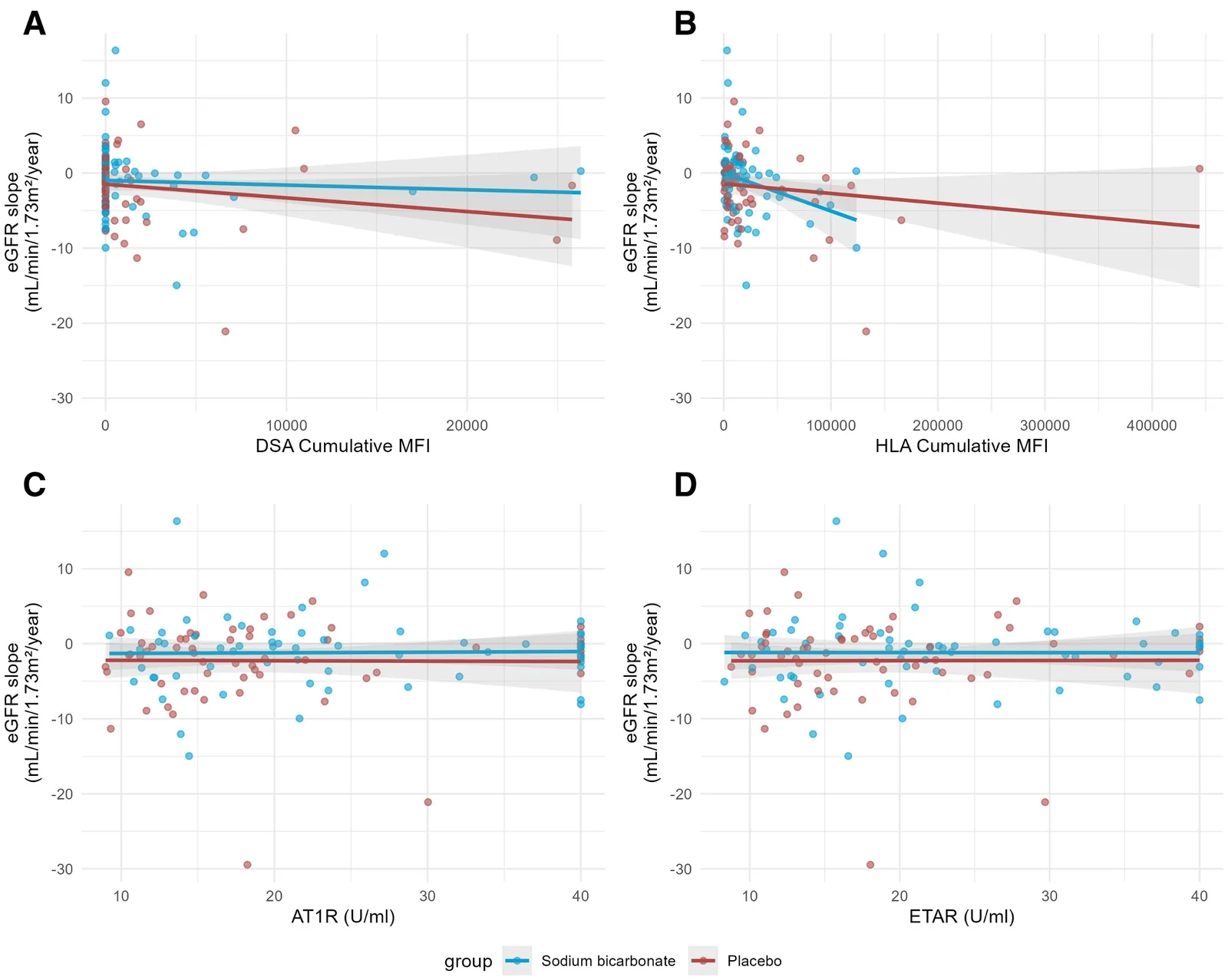
Association between eGFR slope (A–D) and baseline antibody levels. AT1R, angiotensin II type 1 receptor; DSA, donor-specific antibody; eGFR, estimated glomerular filtration rate; ETAR, endothelin type A receptor; MFI, mean fluorescence intensity.

#### Acid-base Status and Immunosuppressive Therapy

As shown in Figure 3, serum bicarbonate and blood pH differed between groups throughout follow-up, indicating adherence to the intervention. Mean serum bicarbonate was 22.8 mmol/L (SD 2.4) in the SB group versus 21.1 mmol/L (SD 2.3) with placebo (*P* < 0.001), yielding a mean difference of 1.6 mmol/L (95% CI, 1.0–2.3) from months 3 to 24. Mean blood pH was 7.39 (SD 0.05) versus 7.36 (SD 0.05) (*P* < 0.001), with a mean difference of 0.03 (95% CI, 0.01–0.04).

**FIGURE 3.**
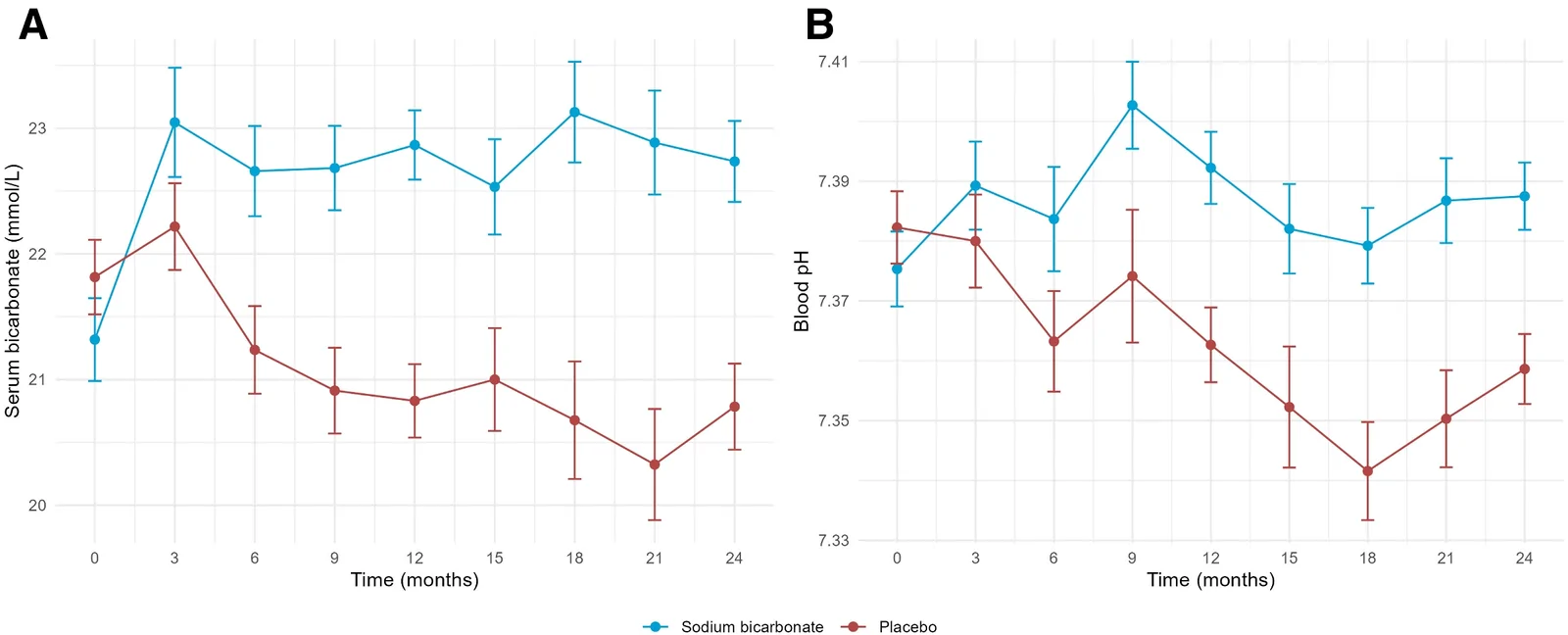
Course of serum bicarbonate (A) and blood pH (B), stratified by treatment group.

The distribution of immunosuppressive agents was comparable between groups and remained stable throughout the study (Figure 4). Mean (SD) trough levels over the study period were similar in the SB and placebo groups for tacrolimus (5.85 [1.80] versus 5.98 [2.63]), cyclosporin (64.5 [27.8] versus 66.7 [56.6]), and mTOR inhibitors (5.67 [1.91] versus 5.14 [1.14]). Mean (SD) and median daily doses were similar between the SB and placebo groups: mycophenolate mofetil, 1614.1 (423.0) and 1500.0 mg/d versus 1571.2 (414.6) and 1500.0 mg/d (*P* = 0.6); mycophenolic acid, 1089.6 (350.8) and 1080.0 mg/d versus 935.3 (238.3) and 1003.5 mg/d (*P* = 0.13); azathioprine, 69.2 (21.1) and 75.0 mg/d versus 78.2 (32.1) and 83.8 mg/d (*P* = 0.4); and corticosteroids, 15.3 (23.0) and 6.3 mg/d versus 8.7 (10.5) and 5.0 mg/d (*P* = 0.088), respectively.

**FIGURE 4.**
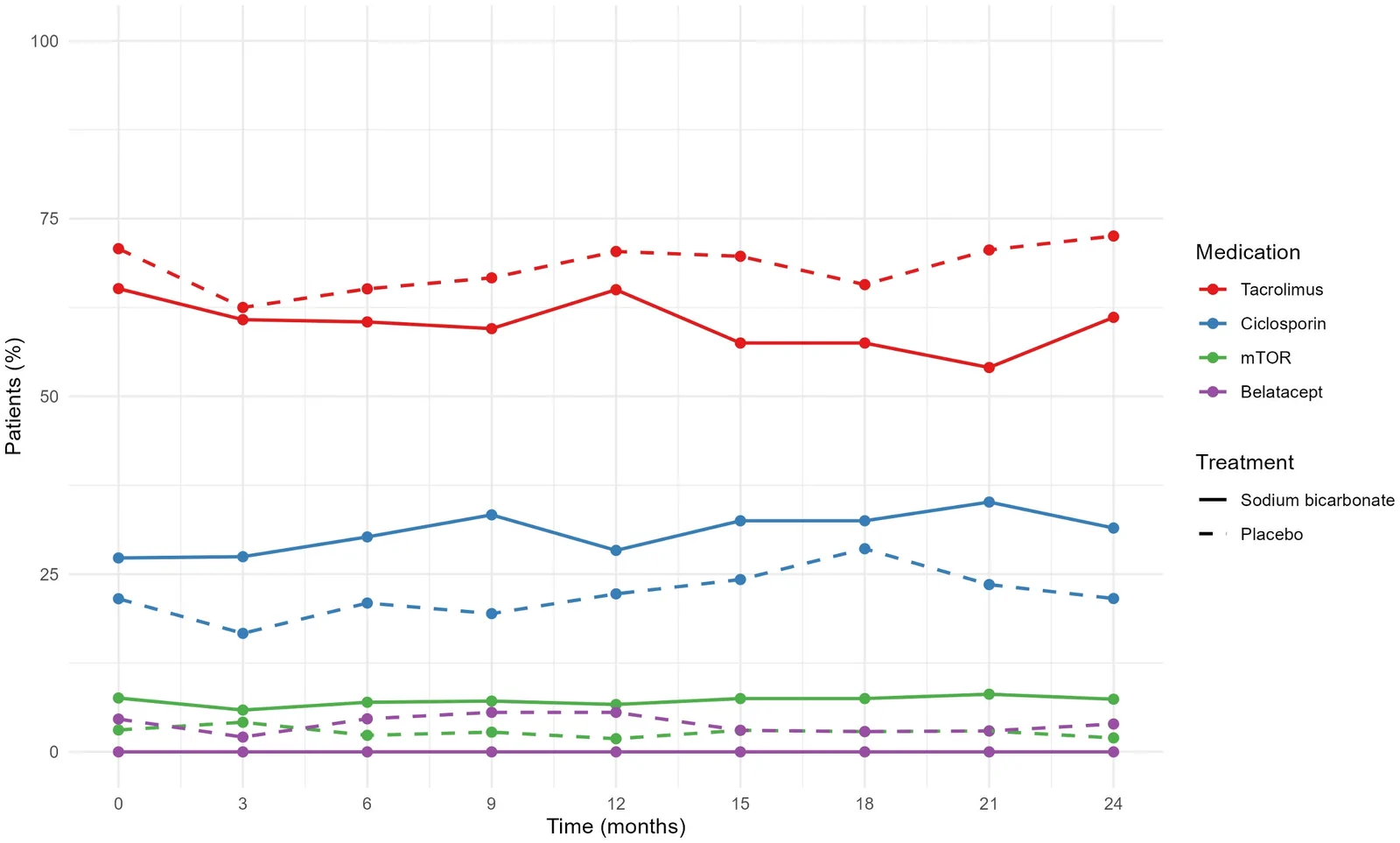
Immunosuppressive therapy over time, stratified by treatment group. mTOR, mechanistic (formerly mammalian) target of rapamycin.

## DISCUSSION

To our knowledge, this is the first study to evaluate SB treatment with regard to non-HLA autoantibodies and HLA antibodies in KTRs with MA. By leveraging data from an RCT, we were able to explore a clinically relevant and biologically plausible hypothesis within a robust methodological framework. Overall, this post hoc analysis provides no evidence to suggest that SB treatment influences non-HLA autoantibody levels (AT1R- and ETAR-Abs) or the cumulative MFI of HLA antibodies, including the cumulative MFI of DSA or their new-onset formation, over 24 mo in long-term KTRs with MA.

Given that MA has been linked to inflammation, endothelial dysfunction, and activation of the renin–angiotensin–aldosterone system as well as ET-1 signaling,^8,11,14,19-21^ a biological connection between MA and both non-HLA autoantibodies (AT1R- and ETAR-Abs) and HLA antibodies in KTRs appears plausible. Experimental data indicate that extracellular acidosis and MA can modulate immune responses, including pH-dependent activation of nuclear factor kappa B and altered production of proinflammatory mediators.^33,34^ In addition, proton-sensing receptors such as GPR4 provide mechanistic support for acid-induced endothelial activation.^13^ Agonistic non-HLA autoantibodies targeting AT1R, often coexisting with ETAR-Abs, promote endothelial activation and proinflammatory signaling, including nuclear factor kappa B–mediated chemokine expression, contributing to microvascular and complement-independent graft injury.^6,35^ As MA may amplify ET-1/ETAR and renin–angiotensin–aldosterone system/AT1R pathways implicated in vascular transplant injury,^8^ untreated MA has been proposed as a potentially modifiable risk factor. Together, these converging pathways support the hypothesis that MA may enhance antibody-mediated vascular injury. However, our exploratory findings do not provide evidence for a measurable effect of MA correction on non-HLA autoantibody or HLA antibody formation, helping to evaluate this hypothesis in a clinical setting.

Non-HLA immunity has increasingly been recognized as a relevant contributor to allograft injury. Early evidence emerged from studies in HLA-identical sibling transplants, demonstrating that graft loss may occur independently of HLA incompatibility.^36^ Subsequently, Dragun et al^35^ identified autoantibodies against the AT1R as mediators of vascular rejection, thereby expanding the concept of antibody-mediated injury beyond classical HLA alloimmunity. Subsequent work identified autoantibodies directed against the ETAR and reported associations with endothelial activation and vascular lesions.^37-39^

Collectively, these findings have established the concept of non-HLA antibody–associated vascular injury in kidney transplantation.^6,40^ A meta-analysis including over 4000 recipients demonstrated significantly increased risks of AMR and graft loss in patients with AT1R-Abs,^41^ with additional evidence supporting a pathogenic role for ETAR-Ab and other non-HLA antibodies for rejection and graft dysfunction.^42^ AT1R- and ETAR-Abs have been shown to be associated with a cytokine profile enriched for a type 2 immune response.^43^ Importantly, while non-HLA autoantibodies may partially account for MVI in the absence of detectable HLA antibodies, evidence suggests that non-HLA and HLA antibody systems are complementary and may interact synergistically in mediating allograft injury. Experimental data further suggest that AT1R-Abs can enhance endothelial HLA expression, thereby potentially amplifying alloimmune responses.^44^ To date, evidence remains insufficient to determine whether isolated, non–event-driven late post-transplant AT1R-/ETAR-Ab positivity carries the same prognostic significance as pretransplant or early post-transplant antibody positivity.

In this context, the findings from our post hoc analysis are notable, as the course of non-HLA autoantibody levels or cumulative MFI of HLA antibodies, including DSA and their new-onset formation were comparable between SB and placebo-treated KTRs. Given the established role of these antibody systems in graft injury, our results suggest that MA is unlikely to be a major driver of antibody formation in stable long-term KTRs.

An important question is why treatment with SB appeared to have no major effect on non-HLA autoantibody or HLA antibody outcomes in this exploratory setting. It remains unclear whether earlier initiation of SB treatment after transplantation, rather than treatment at a later stage, more severe MA, more intensive alkali therapy, or a higher degree of immunization would have yielded different results. Although available data suggest links between acidosis, endothelial dysfunction, and inflammatory activation, the overall evidence remains limited. As MA is often more pronounced in KTRs than in patients with nontransplant chronic kidney disease, partly due to CNI-induced renal tubular acidosis,^9,45,46^ its potential proinflammatory effects may be particularly relevant in the transplant setting. However, many experimental studies induced inflammatory responses using marked acidification (pH 6.5–7.0), conditions substantially more severe than those observed in our cohort or routine clinical practice, limiting direct translational applicability.^33^ In addition, concomitant immunosuppression may attenuate inflammatory signaling and B-cell activation, potentially masking any effect of MA or its correction on antibody production.

Independent of SB treatment, non–CNI-based immunosuppression was associated with lower AT1R-Ab levels, consistent with a potential role of mTOR signaling in antibody-mediated endothelial injury.^47^ However, the small sample size, confidence intervals including the null, and lack of a corresponding association for ETAR-Abs preclude firm conclusions.

In addition to study assignment, male KTRs had higher levels of both non-HLA autoantibodies (AT1R- and ETAR-Abs), possibly reflecting residual confounding. Current KTx literature does not support a consistent sex-specific pattern, although 1 adult study reported an association between male sex and ETAR-Ab positivity.^37,48,49^ In interaction models, male sex was associated with higher 24-mo AT1R-Ab levels and a similar nonsignificant trend for ETAR-Abs. In sex-stratified analyses, SB had no significant treatment effect in either sex (**Table S4, SDC,** https://links.lww.com/TXD/A918).

As previously reported,^6,50^ AT1R- and ETAR-Abs were associated, suggesting potential synergistic effects. In pediatric KTRs, coexistence of both autoantibodies was linked to a >30% decline in eGFR, but not rejection or allograft loss.^50^ However, this finding was not replicated in our cohort, possibly due to differences in patient characteristics and the interval between transplantation and antibody assessment. While cumulative DSA and HLA antibody MFI were associated with eGFR slope, no such association was observed for non-HLA autoantibodies, alone or in combination. This is consistent with the uncertain clinical relevance of non-HLA autoantibodies and the lack of current recommendations for routine testing or treatment.^51^ Overall, within the transplant setting, MA appears to have limited relevance compared with other determinants of endothelial activation and non-HLA autoantibody and HLA antibody burden and may therefore not exert a clinically meaningful effect on the development of these antibodies. While small effects cannot be excluded, our exploratory findings do not support a substantial role of MA in this context.

Our findings are clinically relevant, as they suggest that in the population studied, SB treatment does not substantially influence immunization with non-HLA autoantibody or HLA antibodies and does not provide evidence for a biologically plausible but previously untested hypothesis linking MA to this humoral immune response. Furthermore, non–event-driven late post-transplant AT1R-/ETAR-Ab positivity in KTRs with MA was not associated with eGFR decline, arguing against routine testing and treatment in this setting.

This study has several limitations. First, given its exploratory design and modest sample size, the findings should be considered hypothesis-generating and do not permit definitive conclusions. In particular, the occurrence of de novo DSA during the study period was infrequent, limiting our ability to draw definitive conclusions regarding this outcome. The analysis was limited to 131 participants from the Zurich cohort of the PTS, reflecting the substantial resource requirements associated with non-HLA autoantibody and HLA antibody analyses. Nevertheless, the findings are derived from high-quality randomized data, and the absence of consistent trends across multiple complementary analyses argues against a missed clinically meaningful effect. Second, generalizability may be limited by the predominantly Caucasian study population and the single-center design. Conversely, the single-center setting may strengthen internal validity by ensuring consistent clinical management. Third, thresholds for non-HLA autoantibodies remain poorly defined and have not been established against clinical outcomes in large prospective studies, which limits the interpretation of these measurements.^5,6,52,53^ Nevertheless, thresholds of 10 U/mL for ETAR-Abs and 10 and 17 U/mL for AT1R-Abs appear clinically relevant. To facilitate comparison with the existing literature, we therefore reported both cutoff values for both autoantibodies. Fourth, our analysis was limited to the humoral arm of the immune system and did not assess SB and cellular immune responses. Furthermore, an effect of sodium, as a component of the active alkali treatment, cannot be excluded, as evidence suggests that salt may influence both the adaptive and innate immune systems.^54-57^

The study also has important strengths. It was based on a prospective, randomized, placebo-controlled, single-blinded trial, providing a robust framework for hypothesis testing. Patients were well characterized, and serial samples together with a trial biobank enabled comprehensive assessment of non-HLA autoantibodies and HLA antibodies using validated assays. Analyses were performed in central laboratories, ensuring consistency and comparability. Separation in serum bicarbonate levels was maintained throughout follow-up, indicating good adherence, while immunosuppressive therapy remained comparable between groups. Finally, the 2-y follow-up was considered sufficient to study continuous SB treatment with regard to antibody profiles.

In conclusion, this post hoc analysis provides no evidence to suggest that SB influences non-HLA autoantibodies (AT1R- and ETAR-Abs) or HLA antibodies, including DSA, in long-term KTRs with MA over 24 mo of treatment. While this study provides RCT-derived insights into humoral immunity and SB treatment in KTRs with MA, its exploratory nature warrants confirmation in adequately powered prospective studies.

## ACKNOWLEDGMENTS

The authors thank the patients for their participation; the trial center investigators and study coordinators for their substantial contribution to the conduct of the trial; and the trial monitors for their thorough review work. Furthermore, the authors thank the Department of Immunology, University Hospital Zurich, in particular Zera Gündüz and the laboratory staff (Department of Immunology, University Hospital Zurich), Elisabeth Samland (Division of Nephrology, University Hospital Zurich), as well as Udo Schnitzbauer (Institute of Physiology, University of Zurich), for processing the samples for the antibody analyses of this study.

## Supplementary Material

## References

[1] D’Costa MR Bentall A Denic A. Chronic histologic changes are present regardless of HLA mismatches: evidence from HLA-Identical living donor kidney transplants. Transplantation. 2021;105:e244–e256. 10.1097/TP.0000000000003579 33315759

[2] Stegall MD Cornell LD Park WD. Renal allograft histology at 10 years after transplantation in the tacrolimus era: evidence of pervasive chronic injury. Am J Transplant. 2018;18:180–188. 10.1111/ajt.14431 28710896

[3] Lebraud E Eloudzeri M Rabant M. Microvascular inflammation of the renal allograft: a reappraisal of the underlying mechanisms. Front Immunol. 2022;13:864730. 10.3389/fimmu.2022.864730 PMC8980419 35392097

[4] Naesens M Roufosse C Haas M. The Banff 2022 Kidney Meeting Report: reappraisal of microvascular inflammation and the role of biopsy-based transcript diagnostics. Am J Transplant. 2024;24:338–349. 10.1016/j.ajt.2023.10.016 38032300

[5] Martin PJP Willicombe M Roufosse C. Angiotensin II type-1 receptor antibody in solid organ transplantation—is it time to test? Transpl Int. 2024;37:13280. 10.3389/ti.2024.13280 PMC11598415 39606688

[6] Lefaucheur C Louis K Philippe A. The emerging field of non-human leukocyte antigen antibodies in transplant medicine and beyond. Kidney Int. 2021;100:787–798. 10.1016/j.kint.2021.04.044 34186057

[7] Harmacek D Weidmann L Rho E. Non-human leukocyte antigen autoantibodies-anti-angiotensin II type 1 receptor and endothelin type A receptor antibodies-in the diagnosis of antibody-mediated rejection: biopsy-based transcript diagnostics perspective. Am J Transplant. 2025;25:2320–2328. 10.1016/j.ajt.2025.07.2476 40738402

[8] Kuhn C Mohebbi N Ritter A. Metabolic acidosis in chronic kidney disease: mere consequence or also culprit? Pflugers Arch. 2024;476:579–592. 10.1007/s00424-024-02912-5 PMC11006741 38279993

[9] Messa PG Alfieri C Vettoretti S. Metabolic acidosis in renal transplantation: neglected but of potential clinical relevance. Nephrol Dial Transplant. 2016;31:730–736. 10.1093/ndt/gfv098 25934992

[10] Ritter A Mohebbi N. Causes and consequences of metabolic acidosis in patients after kidney transplantation. Kidney Blood Press Res. 2020;45:792–801. 10.1159/000510158 33040055

[11] Kendrick J Shah P Andrews E. Effect of treatment of metabolic acidosis on vascular endothelial function in patients with CKD: a pilot randomized cross-over study. Clin J Am Soc Nephrol. 2018;13:1463–1470. 10.2215/CJN.00380118 PMC6218835 30237219

[12] Chen B Liu J Ho TT. ERK-mediated NF-κB activation through ASIC1 in response to acidosis. Oncogenesis. 2016;5:e279. 10.1038/oncsis.2016.81 PMC5177778 27941930

[13] Dong L Li Z Leffler NR. Acidosis activation of the proton-sensing GPR4 receptor stimulates vascular endothelial cell inflammatory responses revealed by transcriptome analysis. PLoS One. 2013;8:e61991. 10.1371/journal.pone.0061991 PMC3628782 23613998

[14] Kraut JA Madias NE. Adverse effects of the metabolic acidosis of chronic kidney disease. Adv Chronic Kidney Dis. 2017;24:289–297. 10.1053/j.ackd.2017.06.005 29031355

[15] Nath KA Hostetter MK Hostetter TH. Pathophysiology of chronic tubulo-interstitial disease in rats. Interactions of dietary acid load, ammonia, and complement component C3. J Clin Invest. 1985;76:667–675. 10.1172/JCI112020 PMC423874 2993363

[16] Ori Y Zingerman B Bergman M. The effect of sodium bicarbonate on cytokine secretion in CKD patients with metabolic acidosis. Biomed Pharmacother. 2015;71:98–101. 10.1016/j.biopha.2015.02.012 25960222

[17] Knopf P Stowbur D Hoffmann SHL. Acidosis-mediated increase in IFN-γ-induced PD-L1 expression on cancer cells as an immune escape mechanism in solid tumors. Mol Cancer. 2023;22:207. 10.1186/s12943-023-01900-0 PMC10722725 38102680

[18] Cehakova M Ivanisova D Strecanska M. Rheumatoid synovial fluid and acidic extracellular pH modulate the immunomodulatory activity of urine-derived stem cells. Int J Mol Sci . 2023;24:15856. 10.3390/ijms242115856 PMC10648750 37958839

[19] Henger A Tutt P Riesen WF. Acid-base and endocrine effects of aldosterone and angiotensin II inhibition in metabolic acidosis in human patients. J Lab Clin Med. 2000;136:379–389. 10.1067/mlc.2000.110371 11079465

[20] Jones GV Wall BM Williams HH. Modulation of plasma aldosterone by physiological changes in hydrogen ion concentration. Am J Physiol. 1992;262:R269–R275. 10.1152/ajpregu.1992.262.2.R269 1311533

[21] Ponda MP Hostetter TH. Aldosterone antagonism in chronic kidney disease. Clin J Am Soc Nephrol. 2006;1:668–677. 10.2215/CJN.00120106 17699271

[22] Barton M Shaw S d’Uscio LV. Angiotensin II increases vascular and renal endothelin-1 and functional endothelin converting enzyme activity in vivo: role of ETA receptors for endothelin regulation. Biochem Biophys Res Commun. 1997;238:861–865. 10.1006/bbrc.1997.7394 9325182

[23] Phisitkul S Hacker C Simoni J. Dietary protein causes a decline in the glomerular filtration rate of the remnant kidney mediated by metabolic acidosis and endothelin receptors. Kidney Int. 2008;73:192–199. 10.1038/sj.ki.5002647 17978813

[24] Wesson DE Buysse JM Bushinsky DA. Mechanisms of metabolic acidosis-induced kidney injury in chronic kidney disease. J Am Soc Nephrol. 2020;31:469–482. 10.1681/ASN.2019070677 PMC7062220 31988269

[25] Imenez Silva PH Wiegand A Daryadel A. Acidosis and alkali therapy in patients with kidney transplant is associated with transcriptional changes and altered abundance of genes involved in cell metabolism and acid-base balance. Nephrol Dial Transplant. 2021;36:1806–1820. 10.1093/ndt/gfab210 34240183

[26] Siriteanu L Covic AS Namolovan C. Histological features of kidney allograft biopsies according to metabolic acidosis status: a biopsy-based single-center observational study. Life (Basel). 2026;16:97. 10.3390/life16010097 PMC12843253 41598252

[27] Mohebbi N Ritter A Wiegand A. Sodium bicarbonate for kidney transplant recipients with metabolic acidosis in Switzerland: a multicentre, randomised, single-blind, placebo-controlled, phase 3 trial. Lancet. 2023;401:557–567. 10.1016/S0140-6736(22)02606-X 36708734

[28] Wiegand A Ritter A Graf N. Preservation of kidney function in kidney transplant recipients by alkali therapy (Preserve-Transplant Study): rationale and study protocol. BMC Nephrol. 2018;19:177. 10.1186/s12882-018-0956-8 PMC6043955 30001705

[29] CellTrend GmbH. EIA zur quantitativen Bestimmung von humanen anti-Angiotensin II-Rezeptor Typ 1-Antikörpern. CellTrend GmbH. Available at https://www.celltrend.de/wp-content/uploads/2024/09/12000-AT1R-Gebrauchsinformation-deutsch-V06-07_24.pdf. Accessed September 6, 2026.

[30] CellTrend GmbH. EIA zur quantitativen Bestimmung von humanen anti-Endothelin-Rezeptor A-Antikörpern. CellTrend GmbH. Available at https://www.celltrend.de/wp-content/uploads/2024/09/12100-ETAR-Gebrauchsinformation-deutsch-V06-08_24.pdf. Accessed September 6, 2026.

[31] Levey AS Stevens LA Schmid CH; CKD-EPI (Chronic Kidney Disease Epidemiology Collaboration). A new equation to estimate glomerular filtration rate. Ann Intern Med. 2009;150:604–612. 10.7326/0003-4819-150-9-200905050-00006 PMC2763564 19414839

[32] R Core Team. R: a language and environment for statistical computing. Available at: https://www.R-project.org/. Accessed June 25, 2026.

[33] de Nadai TR de Nadai MN Albuquerque AAS. Metabolic acidosis treatment as part of a strategy to curb inflammation. Int J Inflam. 2013;2013:601424. 10.1155/2013/601424 PMC3690240 23841017

[34] Kellum JA Song M Li J. Science review: extracellular acidosis and the immune response: clinical and physiologic implications. Crit Care. 2004;8:331–336. 10.1186/cc2900 PMC1065014 15469594

[35] Dragun D Müller DN Bräsen JH. Angiotensin II type 1-receptor activating antibodies in renal-allograft rejection. N Engl J Med. 2005;352:558–569. 10.1056/NEJMoa035717 15703421

[36] Opelz G; Collaborative Transplant Study. Non-HLA transplantation immunity revealed by lymphocytotoxic antibodies. Lancet. 2005;365:1570–1576. 10.1016/S0140-6736(05)66458-6 15866311

[37] Banasik M Boratyńska M Kościelska-Kasprzak K. The impact of non-HLA antibodies directed against endothelin-1 type A receptors (ETAR) on early renal transplant outcomes. Transpl Immunol. 2014;30:24–29. 10.1016/j.trim.2013.10.007 24184747

[38] Sigdel TK Li L Tran TQ. Non-HLA antibodies to immunogenic epitopes predict the evolution of chronic renal allograft injury. J Am Soc Nephrol. 2012;23:750–763. 10.1681/ASN.2011060596 22302197

[39] Nowańska K Wiśnicki K Kuriata-Kordek M. The role of endothelin II type A receptor (ETAR) in transplant injury. Transpl Immunol. 2022;70:101505. 10.1016/j.trim.2021.101505 34793957

[40] Philogene MC Johnson T Vaught AJ. Antibodies against angiotensin II type 1 and endothelin A receptors: relevance and pathogenicity. Hum Immunol. 2019;80:561–567. 10.1016/j.humimm.2019.04.012 PMC8015780 31010696

[41] Kang ZY Liu C Liu W. Effect of anti-angiotensin II type 1 receptor antibodies on the outcomes of kidney transplantation: a systematic review and meta-analysis. Nephrol Dial Transplant. 2022;37:1171–1180. 10.1093/ndt/gfab344 34865146

[42] Fichtner A Süsal C Höcker B. Association of non-HLA antibodies against endothelial targets and donor-specific HLA antibodies with antibody-mediated rejection and graft function in pediatric kidney transplant recipients. Pediatr Nephrol. 2021;36:2473–2484. 10.1007/s00467-021-04969-1 PMC8260519 33759004

[43] Pearl MH Chen L Grogan T. Antibodies to angiotensin II type 1 receptor and endothelin type A receptor are associated with cytokine production enriched for type 2 immune response and antibody production in pediatric kidney transplant recipients. Am J Transplant. 2025;25:2329–2344. 10.1016/j.ajt.2025.07.2486 PMC12704516 40774357

[44] Dragun D Catar R Philippe A. Non-HLA antibodies against endothelial targets bridging allo- and autoimmunity. Kidney Int. 2016;90:280–288. 10.1016/j.kint.2016.03.019 27188505

[45] Mohebbi N Mihailova M Wagner CA. The calcineurin inhibitor FK506 (tacrolimus) is associated with transient metabolic acidosis and altered expression of renal acid-base transport proteins. Am J Physiol Renal Physiol. 2009;297:F499–F509. 10.1152/ajprenal.90489.2008 19439519

[46] Watanabe S Tsuruoka S Vijayakumar S. Cyclosporin A produces distal renal tubular acidosis by blocking peptidyl prolyl *cis-trans* isomerase activity of cyclophilin. Am J Physiol Renal Physiol. 2005;288:F40–F47. 10.1152/ajprenal.00218.2004 15353404

[47] Catar RA Wischnewski O Chen L. Non-HLA antibodies targeting angiotensin II type 1 receptor and endothelin-1 type A receptors induce endothelial injury via β2-arrestin link to mTOR pathway. Kidney Int. 2022;101:498–509. 10.1016/j.kint.2021.09.029 34757123

[48] Lefaucheur C Viglietti D Bouatou Y. Non-HLA agonistic anti-angiotensin II type 1 receptor antibodies induce a distinctive phenotype of antibody-mediated rejection in kidney transplant recipients. Kidney Int. 2019;96:189–201. 10.1016/j.kint.2019.01.030 31005275

[49] Viboon S Townamchai N Phiancharoen S. Prevalence of antibody against angiotensin II type 1 receptor (AT1R) among Thai kidney transplant patients. Transplant Proc. 2018;50:1310–1315. 10.1016/j.transproceed.2018.03.020 29880351

[50] Pearl MH Chen L ElChaki R. Endothelin type A receptor antibodies are associated with angiotensin II type 1 receptor antibodies, vascular inflammation, and decline in renal function in pediatric kidney transplantation. Kidney Int Rep. 2020;5:1925–1936. 10.1016/j.ekir.2020.09.004 PMC7609952 33163713

[51] Schinstock CA Gebel H Gimferrer I; STAR Working Group Members. Sensitization in organ transplantation: assessment of Risk (STAR) 2025 meeting group report. Am J Transplant. 2026;26:1048–1069. 10.1016/j.ajt.2025.12.286 41519473

[52] Goutaudier V Danger R Catar RA; EU-TRAIN Consortium. Evaluation of non-invasive biomarkers of kidney allograft rejection in a prospective multicenter unselected cohort study (EU-TRAIN). Kidney Int. 2024;106:943–960. 10.1016/j.kint.2024.07.027 39197587

[53] Sorohan BM Ismail G Leca N. Angiotensin II type 1 receptor antibodies in kidney transplantation: an evidence-based comprehensive review. Transplant Rev (Orlando). 2020;34:100573. 10.1016/j.trre.2020.100573 33002671

[54] Staub PS Kronthaler D Wiegand A. Effect of sodium bicarbonate on indicators of volume retention in metabolic acidosis after kidney transplantation: a post-hoc analysis. Kidney Med. 2026;8:101290. 10.1016/j.xkme.2026.101290 PMC13053739 41953463

[55] Evans RDR Antonelou M Henderson S. Emerging evidence of an effect of salt on innate and adaptive immunity. Nephrol Dial Transplant. 2019;34:2007–2014. 10.1093/ndt/gfy362 30521016

[56] Rauf MA Agarwal S Baker RR. Inherited salt retention is associated with increased IL-17 responses and autoimmunity. JCI Insight. 2026;11:e206932. 10.1172/jci.insight.206932 PMC13502187 42467560

[57] Kleinewietfeld M Manzel A Titze J. Sodium chloride drives autoimmune disease by the induction of pathogenic TH17 cells. Nature. 2013;496:518–522. 10.1038/nature11868 PMC3746493 23467095

