# Supplementary material for: Sodium Bicarbonate and Non-HLA Autoantibody or HLA Antibody Trajectories in Long-term Kidney Transplant Recipients With Metabolic Acidosis: A Post Hoc Analysis of the Preserve-Transplant Randomized Controlled Trial
Source: Transplant Direct. 2026 Nov 6;12(12):e2022. doi: 10.1097/TXD.0000000000002022 (PMC13747465; doi:10.1097/TXD.0000000000002022)
Supplement: Supplementary file 1 [file txd-12-e2022-s001.pdf]

Supplemental Material

Sodium bicarbonate and non-HLA auto-antibody or HLA antibody trajectories in long-term kidney transplant recipients with metabolic acidosis: a post-hoc analysis of the Preserve-Transplant randomized controlled trial

Alexander Ritter, MD[1,2], Jakob Nilsson, PhD[3], Erin West, MSc[4], Christian Kuhn, MD[2], Carsten A. Wagner, Prof.[5], Thomas Schachtner, Prof.[1], Nilufar Mohebbi, Prof.[1,6]

Affiliations:

[1]Division of Nephrology, University Hospital Zurich, Zurich, Switzerland;

[2]Division of Nephrology and Transplantation Medicine, HOCH Cantonal Hospital St.Gallen, St. Gallen, Switzerland;

[3]Department of Immunology, University Hospital Zurich, Zurich, Switzerland;

[4]Biostatistics at Clinical Trials Unit, HOCH Cantonal Hospital St.Gallen, St. Gallen, Switzerland;

[5]Institute of Physiology and Zurich Kidney Center, University of Zurich, Zurich, Switzerland;

[6]Praxis und Dialysezentrum Zürich-City AG, Zurich, Switzerland

Contents of Supplemental Material

Supplemental Tables

Table S1. Presence of AT1R and ETAR antibodies at baseline (all subjects with data)
Table S2. Sensitivity Analysis - Absolute levels of AT1R- and ETAR-Abs without 40 U/ml cap
Table S3. Sensitivity Analysis - Linear regression models for 24-month AT1R- and ETAR-Ab levels without 40 U/ml cap
Table S4. Effect modification analyses for treatment effects on 24-month non-HLA auto-antibody levels (directed against AT1R and ETAR)
Table S5. Kinetics of AT1R and ETAR antibodies - Category transitions (pairwise comparisons)
Table S6. Baseline characteristics of patients with improved vs. not improved AT1R antibody titers (baseline to 24 months)
Table S7. Baseline characteristics of patients with improved vs. not improved ETAR antibody titers (baseline to 24 months)

Supplemental Figures

Figure S1. Study Flow Chart showing the Analysis Dataset deriving from the Preserve-Transplant Study.
Figure S2. AT1R Antibody Category Changes (Baseline to 24 Months).
Figure S3. ETAR Antibody Category Changes (Baseline to 24 Months).
Figure S4. Bland-Altman plots of AT1R (A) and ETAR (B) antibody levels.

Table S1: Presence of AT1R and ETAR antibodies at baseline (all subjects with data)

Characteristic	Overall N = 130[1]	Sodium bicarbonate N = 65[1]	Placebo N = 65[1]	p-value[2]
AT1R antibody status				0.15
Normal (< 10 U/ml)	5 (3.8%)	1 (1.5%)	4 (6.2%)	
Risk (10 - < 17 U/ml)	49 (38%)	21 (32%)	28 (43%)	
Positive (≥ 17 U/ml)	76 (58%)	43 (66%)	33 (51%)	
ETAR antibody status				0.14
Normal (< 10 U/ml)	5 (3.8%)	2 (3.1%)	3 (4.6%)	
Risk (10 - < 17 U/ml)	48 (37%)	19 (29%)	29 (45%)	
Positive (≥ 17 U/ml)	77 (59%)	44 (68%)	33 (51%)	
[1]n (%)				
[2]Fisher's exact test				

Table S2: Sensitivity Analysis - Absolute levels of AT1R- and ETAR-Abs without 40 U/ml cap

Characteristic	Overall N = 105[1]	Sodium Bicarbonate N = 54[1]	Placebo N = 51[1]	p-value[2]
Time between measurements (months)	25.4 (3.0)	25.7 (3.0)	25.1 (3.0)	0.13
AT1R Ab at baseline (U/ml)	22 (13)	25 (14)	19 (11)	0.016
AT1R Ab at 24 months (U/ml)	23 (13)	25 (14)	21 (11)	0.061
AT1R Ab change (U/ml)	1 (8)	0 (10)	1 (6)	0.2
ETAR Ab at baseline (U/ml)	22 (12)	25 (13)	19 (10)	0.014
ETAR Ab at 24 months (U/ml)	23 (12)	25 (14)	21 (10)	0.077
ETAR Ab change (U/ml)	1 (9)	1 (10)	2 (6)	0.030
[1]Mean (SD)				
[2]Wilcoxon rank sum test				

Table S3: Sensitivity Analysis - Linear regression models for 24-month AT1R- and ETAR-Ab levels without 40 U/ml cap

	AT1R-Ab at 24 months			ETAR-Ab at 24 months		
Predictor	Beta	95% CI	p-value	Beta	95% CI	p-value
**Baseline Model**						
Sodium bicarbonate	-0.37	-3.56, 2.83	0.82	0.14	-3.07, 3.35	0.93
**Adjusted Model**						
Sodium bicarbonate	-0.14	-3.25, 2.98	0.93	0.39	-2.73, 3.51	0.81
Age (years)	-0.04	-0.15, 0.07	0.48	-0.02	-0.13, 0.08	0.68
Sex (Male)	3.78	0.59, 6.97	0.02	4.6	1.4, 7.81	0.01
Non-CNI based Immunosuppression*	-5.66	-11.39, 0.07	0.05	-3.6	-9.34, 2.13	0.22

*mTOR inhibitors/belatacept

Table S4: Effect modification analyses for treatment effects on 24-month non-HLA auto-antibody levels (directed against AT1R and ETAR)

**Analysis**	**N**	**Beta**	**95% CI**	**p-value**
**Interaction Tests**				
Treatment × Sex (AT1R Ab)	105	-2.87	-7.94, 2.2	0.26
Treatment × Sex (ETAR Ab)	105	-0.66	-5.82, 4.51	0.8
Treatment × Immunosuppression (AT1R Ab)	105	-4.42	-13.6, 4.77	0.34
Treatment × Immunosuppression (ETAR Ab)	105	-0.89	-10.22, 8.43	0.85
**Main Effect of Male Sex**				
AT1R Ab	105	4.31	0.59, 8.03	0.02
ETAR Ab	105	3.42	-0.36, 7.19	0.08
**Sex-Stratified Models (Treatment Effect)**				
AT1R Ab - Males	68	-1.25	-4.11, 1.61	0.39
AT1R Ab - Females	37	2.33	-2.35, 7.01	0.32
ETAR Ab - Males	68	0.14	-2.88, 3.16	0.92
ETAR Ab - Females	37	0.96	-3.76, 5.68	0.68

Table S5: Kinetics of AT1R and ETAR antibodies - Category transitions (pairwise comparisons)

Characteristic	Overall<br>N = 105[1]	Sodium bicarbonate<br>N = 54[1]	Placebo<br>N = 51[1]	p-value[2]
AT1R Ab: Improved	10 (9.5%)	8 (15%)	2 (3.9%)	0.094
AT1R Ab: Stable	79 (75%)	38 (70%)	41 (80%)	0.3
AT1R Ab: Worsened	16 (15%)	8 (15%)	8 (16%)	>0.9
ETAR Ab: Improved	9 (8.6%)	5 (9.3%)	4 (7.8%)	>0.9
ETAR Ab: Stable	74 (70%)	41 (76%)	33 (65%)	0.3
ETAR Ab: Worsened	22 (21%)	8 (15%)	14 (27%)	0.2

[1]n (%)

[2]Fisher's exact test

Table S6: Baseline characteristics of patients with improved vs. not improved AT1R antibody titers (baseline to 24 months)

Characteristic	Improved<br>N = 46[1]	Not improved<br>N = 59[1]	p-value[2]
group			0.12
Sodium bicarbonate	28 (60.9%)	26 (44.1%)	
Placebo	18 (39.1%)	33 (55.9%)	
Age, years	55.7 (14.6)	50.5 (13.7)	0.053
Sex			0.064
Female	21 (45.7%)	16 (27.1%)	
Male	25 (54.3%)	43 (72.9%)	
Cause of end-stage renal disease			0.7
Hereditary	9 (19.6%)	11 (18.6%)	
Glomerular nephritis	19 (41.3%)	22 (37.3%)	
Tubulointerstitial kidney disease	0 (0.0%)	3 (5.1%)	
Diabetic nephropathy	2 (4.3%)	1 (1.7%)	
Hypertensive nephropathy	2 (4.3%)	2 (3.4%)	
Others	14 (30.4%)	20 (33.9%)	
Time since transplantation, years	9.4 (9.0)	9.2 (7.3)	0.8
Repeat transplantation	7 (15.2%)	3 (5.1%)	0.10
Transplant from living donor	18 (39.1%)	30 (50.8%)	0.2

Transplant from deceased donor	28 (60.9%)	29 (49.2%)	0.2
ABOi	2 (4.3%)	0 (0.0%)	0.2
History of transplant rejection	9 (19.6%)	17 (28.8%)	0.4
Donor-specific HLA antibodies (DSA)	21 (45.7%)	21 (35.6%)	0.3
Cumulative MFI of DSA	1,652.2 (3,451.4)	2,369.6 (6,387.4)	0.4
Non-DSA HLA antibodies	46 (100.0%)	59 (100.0%)	>0.9
Cumulative MFI of non-DSA HLA antibodies	33,754.1 (70,148.3)	22,069.7 (32,823.9)	0.2
Primary immunosuppressant			0.4
Tacrolimus	32 (69.6%)	38 (64.4%)	
Ciclosporin	9 (19.6%)	18 (30.5%)	
mTOR inhibitor	4 (8.7%)	2 (3.4%)	
Belatacept	1 (2.2%)	1 (1.7%)	
Mycophenolate mofetil or mycophenolic acid	42 (91.3%)	55 (93.2%)	0.7
Corticosteroids	16 (34.8%)	18 (30.5%)	0.7
Estimated GFR, mL/min/1.73 m²	51.1 (16.8)	51.2 (16.2)	0.7
Serum bicarbonate, mmol/L	21.4 (2.8)	21.8 (2.4)	0.7
Venous blood pH	7.39 (0.04)	7.37 (0.05)	0.045
Missing	6	7	
Urine protein-to-creatinine ratio, mg/g	271.8 (341.5)	198.9 (200.8)	0.3
Urine albumin-to-creatinine ratio, mg/g	157.2 (293.0)	104.4 (162.6)	0.3
[1]n (%); Mean (SD)			
[2]Fisher's exact test; Wilcoxon rank sum test			

Table S7: Baseline characteristics of patients with improved vs. not improved ETAR antibody titers (baseline to 24 months)

group			0.12
Sodium bicarbonate	28 (60.9%)	26 (44.1%)	
Placebo	18 (39.1%)	33 (55.9%)	
Age, years	55.7 (14.6)	50.5 (13.7)	0.053
Sex			0.064
Female	21 (45.7%)	16 (27.1%)	

Male	25 (54.3%)	43 (72.9%)	
Cause of end-stage renal disease			0.7
Hereditary	9 (19.6%)	11 (18.6%)	
Glomerular nephritis	19 (41.3%)	22 (37.3%)	
Tubulointerstitial kidney disease	0 (0.0%)	3 (5.1%)	
Diabetic nephropathy	2 (4.3%)	1 (1.7%)	
Hypertensive nephropathy	2 (4.3%)	2 (3.4%)	
Others	14 (30.4%)	20 (33.9%)	
Time since transplantation, years	9.4 (9.0)	9.2 (7.3)	0.8
Repeat transplantation	7 (15.2%)	3 (5.1%)	0.10
Transplant from living donor	18 (39.1%)	30 (50.8%)	0.2
Transplant from deceased donor	28 (60.9%)	29 (49.2%)	0.2
ABOi	2 (4.3%)	0 (0.0%)	0.2
History of transplant rejection	9 (19.6%)	17 (28.8%)	0.4
Donor-specific HLA antibodies (DSA)	21 (45.7%)	21 (35.6%)	0.3
Cumulative MFI of DSA	1,652.2 (3,451.4)	2,369.6 (6,387.4)	0.4
Non-DSA HLA antibodies	46 (100.0%)	59 (100.0%)	>0.9
Cumulative MFI of non-DSA HLA antibodies	33,754.1 (70,148.3)	22,069.7 (32,823.9)	0.2
Primary immunosuppressant			0.4
Tacrolimus	32 (69.6%)	38 (64.4%)	
Ciclosporin	9 (19.6%)	18 (30.5%)	
mTOR inhibitor	4 (8.7%)	2 (3.4%)	
Belatacept	1 (2.2%)	1 (1.7%)	
Mycophenolate mofetil or mycophenolic acid	42 (91.3%)	55 (93.2%)	0.7
Corticosteroids	16 (34.8%)	18 (30.5%)	0.7
Estimated GFR, mL/min/1.73 m^2	51.1 (16.8)	51.2 (16.2)	0.7
Serum bicarbonate, mmol/L	21.4 (2.8)	21.8 (2.4)	0.7
Venous blood pH	7.39 (0.04)	7.37 (0.05)	0.045
Missing	6	7	
Urine protein-to-creatinine ratio, mg/g	271.8 (341.5)	198.9 (200.8)	0.3
Urine albumin-to-creatinine ratio, mg/g	157.2 (293.0)	104.4 (162.6)	0.3

[1]n (%); Mean (SD)
[2]Fisher's exact test; Wilcoxon rank sum test

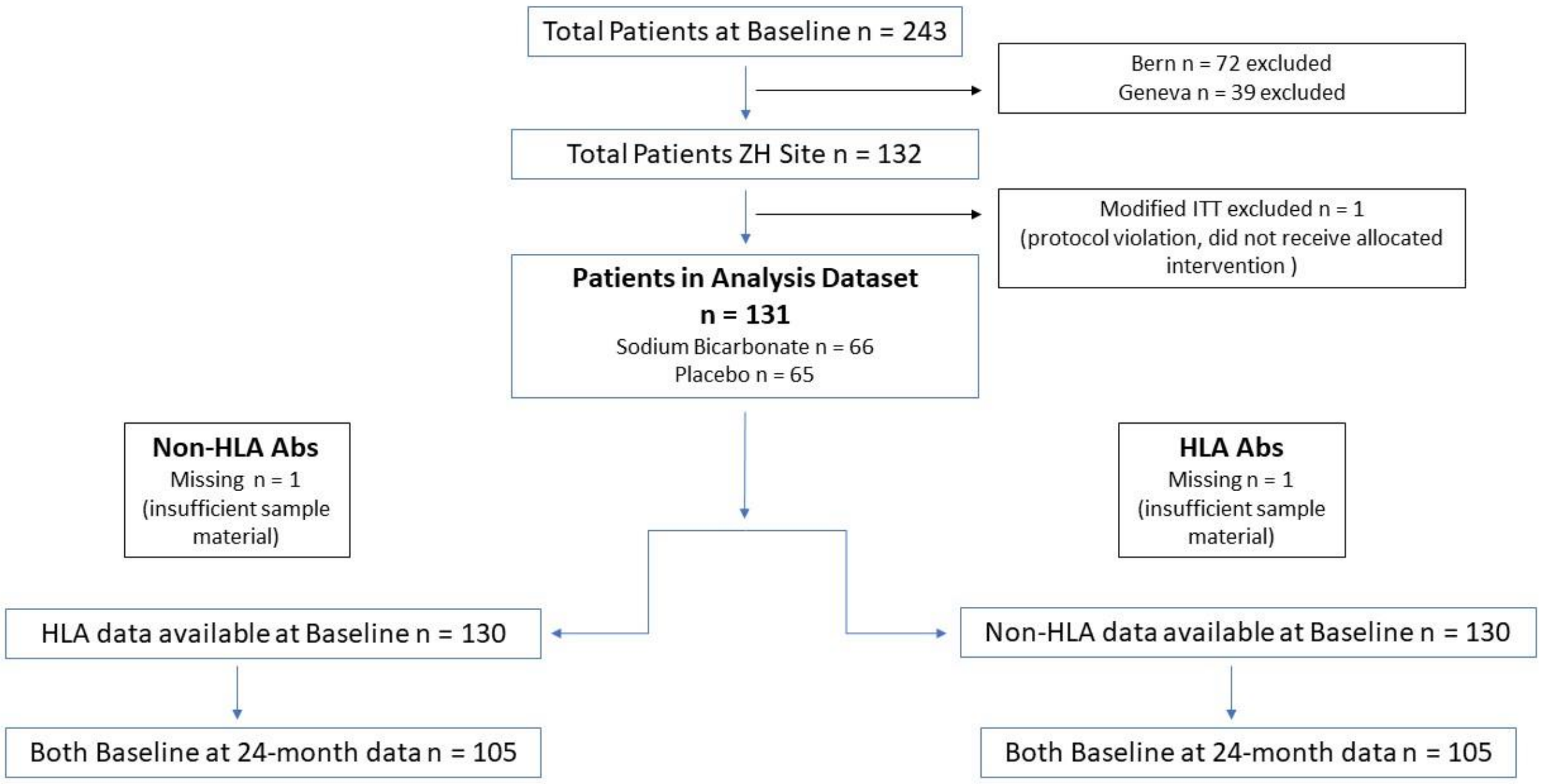

Figure S1: Study Flow Chart showing the Analysis Dataset deriving from the Preserve-Transplant Study. 105 subjects finished the trial regularly at 24 months.

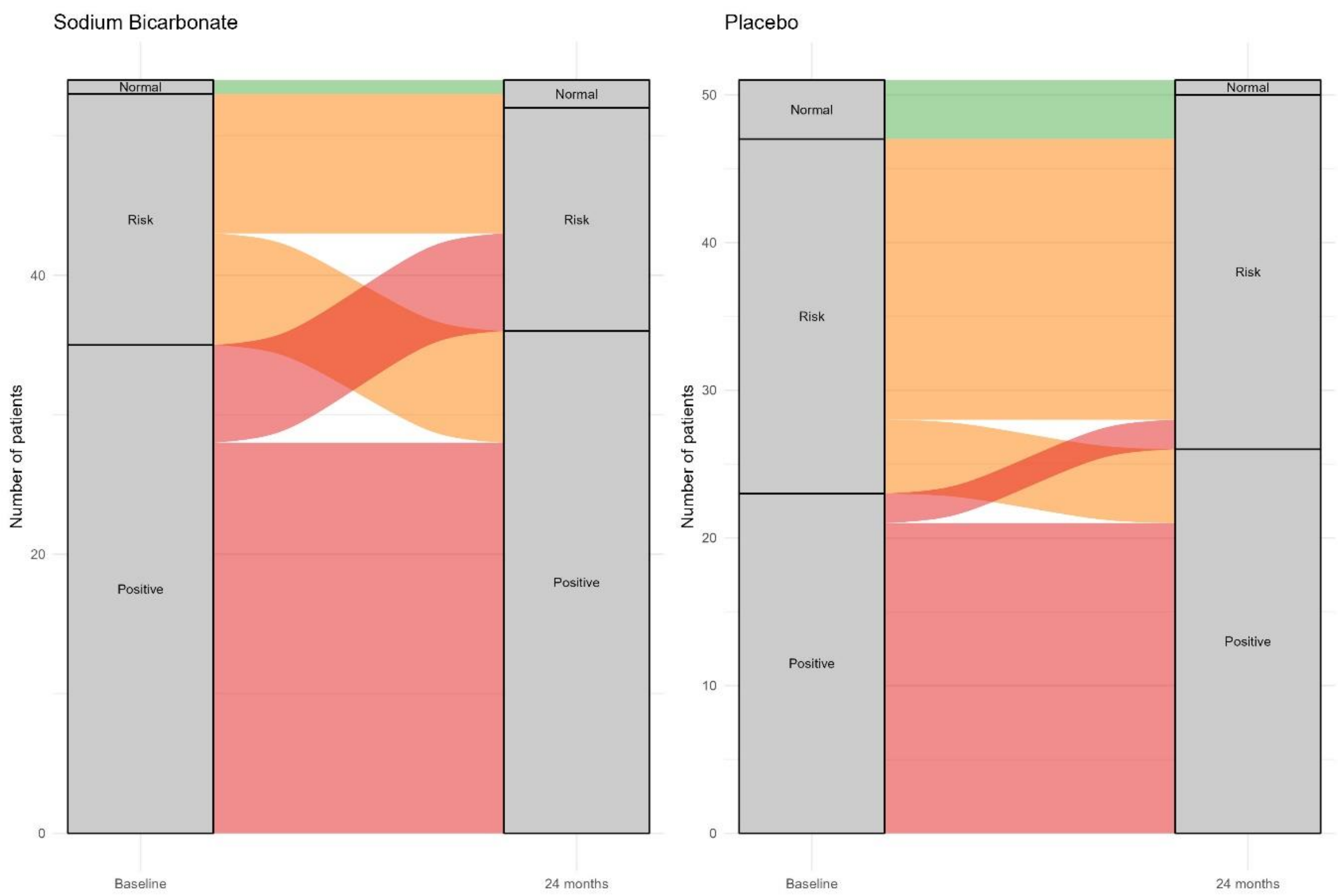

Figure S2: AT1R Antibody Category Changes (Baseline to 24 Months). Transitions for the non-HLA auto-antibody directed against AT1R from baseline to 24 months, stratified by treatment group. Categories are defined as Normal (<10 U/ml), Risk (10 to <17 U/ml), and Positive (≥17 U/ml).

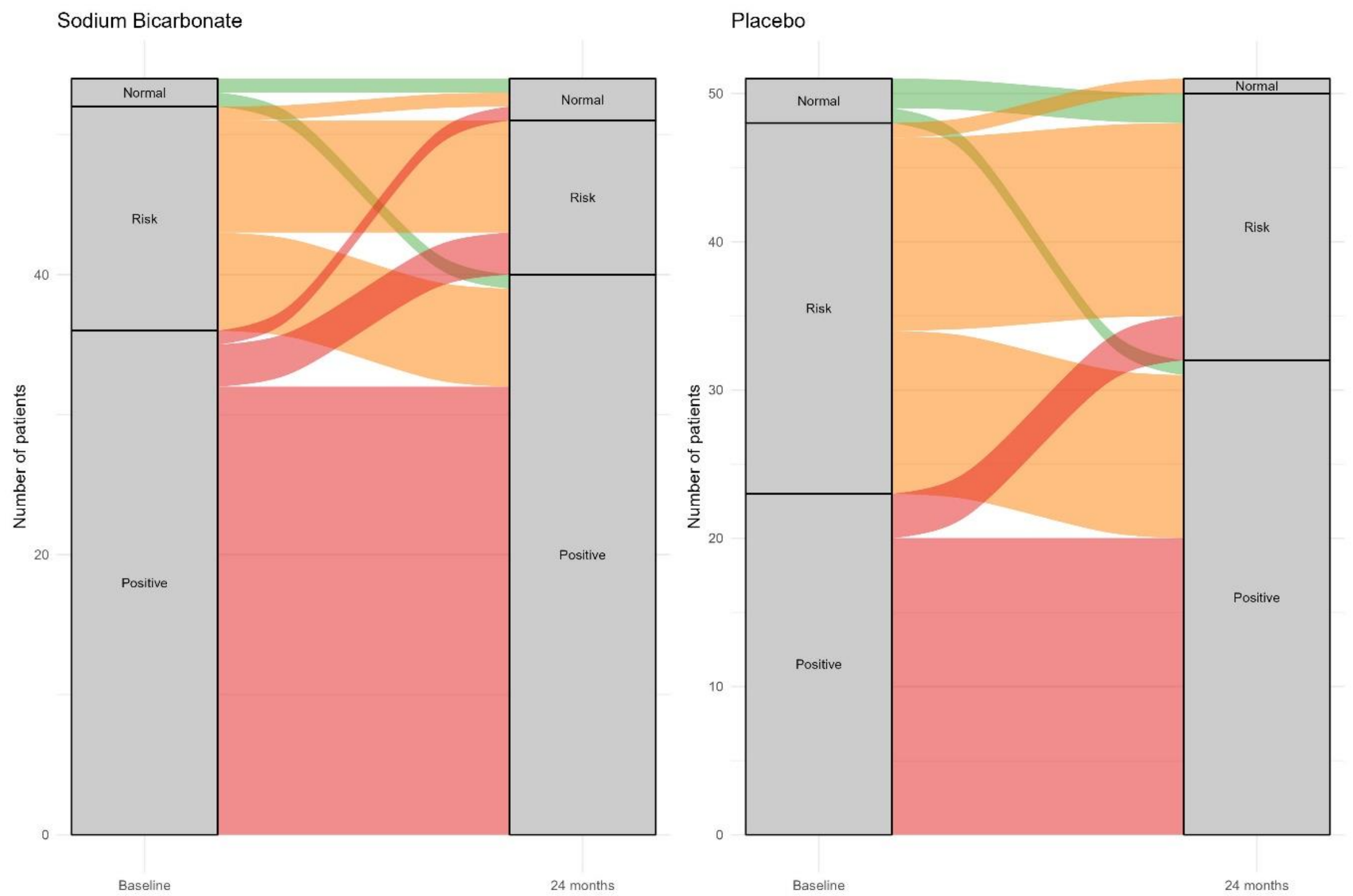

Figure S3: ETAR Antibody Category Changes (Baseline to 24 Months). Transitions for the non-HLA auto-antibody directed against ETAR from baseline to 24 months, stratified by treatment group. Categories are defined as Normal (<10 U/ml), Risk (10 to <17 U/ml), and Positive (≥17 U/ml).

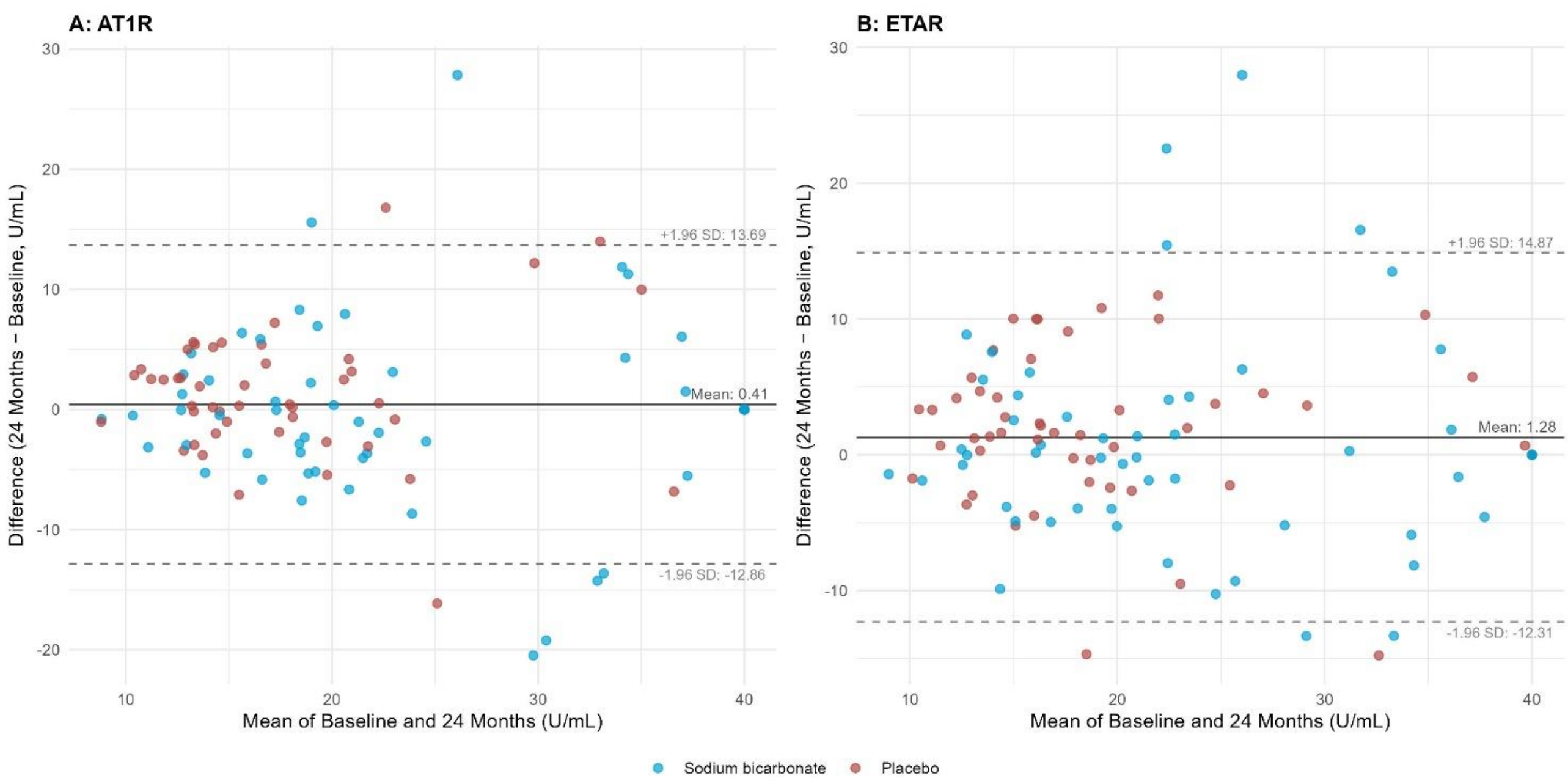

Figure S4: Bland-Altman plots of AT1R (A) and ETAR (B) antibody levels.
